# Identification of candidate genes in ischemic cardiomyopathy by gene expression omnibus database

**DOI:** 10.1186/s12872-020-01596-w

**Published:** 2020-07-06

**Authors:** Haiming Dang, Yicong Ye, Xiliang Zhao, Yong Zeng

**Affiliations:** 1 Department of cardiac surgery, Capital medical university, Beijing Anzhen hospital, Beijing, China; 2 Department of cardiology, Capital medical university, Beijing Anzhen hospital, No.2, Anzhen Road, Chaoyan District, Beijing, 100029 China

**Keywords:** Ischemic cardiomyopathy, Differentially expressed genes, Gene expression omnibus datasets, Integrated analysis

## Abstract

**Background:**

Ischemic cardiomyopathy (ICM) is one of the most usual causes of death worldwide. This study aimed to find the candidate gene for ICM.

**Methods:**

We studied differentially expressed genes (DEGs) in ICM compared to healthy control. According to these DEGs, we carried out the functional annotation, protein-protein interaction (PPI) network and transcriptional regulatory network constructions. The expression of selected candidate genes were confirmed using a published dataset and Quantitative real time polymerase chain reaction (qRT-PCR).

**Results:**

From three Gene Expression Omnibus (GEO) datasets, we acquired 1081 DEGs (578 up-regulated and 503 down-regulated genes) between ICM and healthy control. The functional annotation analysis revealed that cardiac muscle contraction, hypertrophic cardiomyopathy, arrhythmogenic right ventricular cardiomyopathy and dilated cardiomyopathy were significantly enriched pathways in ICM. SNRPB, BLM, RRS1, CDK2, BCL6, BCL2L1, FKBP5, IPO7, TUBB4B and ATP1A1 were considered the hub proteins. PALLD, THBS4, ATP1A1, NFASC, FKBP5, ECM2 and BCL2L1 were top six transcription factors (TFs) with the most downstream genes. The expression of 6 DEGs (MYH6, THBS4, BCL6, BLM, IPO7 and SERPINA3) were consistent with our integration analysis and GSE116250 validation results.

**Conclusions:**

The candidate DEGs and TFs may be related to the ICM process. This study provided novel perspective for understanding mechanism and exploiting new therapeutic means for ICM.

## Background

Ischemic cardiomyopathy (ICM), a common secondary cardiomyopathy, is one of the most common causes of death worldwide [1]. ICM is a special type or later stage of coronary heart disease, caused by coronary atherosclerotic heart disease, but can also be caused by repeated coronary spasm, coronary inflammation and connective tissue disease. ICM also is the most common cause of heart failure and is associated with significant morbidity and mortality [2]. The main pathophysiological features of ischemic cardiomyopathy are left ventricular enlargement, function of ventricular myocardial diastole and contractile decrease, and further development of congestive heart failure. Like other complex diseases, ICM is caused by interactions of environmental factors and genetic. The factors contributing to ICM are complex, including inflammation, microvessels dysfunction, activation of apoptosis and disruption of Ca^2+^ homeostasis [3, 4]. With the emergence of microarray technology analysis, bioinformatics have become most frequently used means to identify potential biomarkers in a variety of diseases [5–7]. It is reported that a lot of fetal and immediate-early genes are deregulated in the ischemic heart [8]. To our knowledge, many researchers have performed global gene expression to obtain key genes in the underlying mechanisms of ICM [9–11]. For example, Qiao et al. reported that differentially expressed genes (DEGs) and transcription factors (TFs) play pivotal roles in ICM progress through regulating gene expression [12]. Li et al. found that the functional annotation and pathway analysis of DEGs was conducive to further studying the interactions between the differentially expressed genes in ICM [13]. Wang et al. found that PHLDA1 might be a novel molecular marker for ICM [14]. Previous studies identified changes in the protein levels of TFs including GATA4, NFAT1, MEF2C, CSX NKX2–5, NF-kB, STAT-3 and AP-1 in cardiomyopathy and cardiopathy model [15–19]. The molecular mechanism of coordinating transcription in ICM has not been completely understood. Therefore, it is essential to find the pathogenic mechanism and develop new diagnostic biomarker.

The appearance of gene microarray data has become an effective means to search DEGs in multiple diseases which help to reveal underlying mechanisms. Genes that cause complex diseases are always involved in common biological processes in various biological networks [20, 21]. A comprehensive understanding of disease can be improved by analyzing the biological data of the network module.

Here, the integrated analysis of multiple GEO datasets was performed to identify DEGs between ICM and healthy control. The bioinformatics methods was applied to obtain the ICM related pathways and TFs. The purpose of our study is to better understand the molecular events and pathways of ICM and to develop new therapeutic means for ICM.

## Methods

### The analysis of microarray data

The expression profile of ICM and healthy control were downloaded from GEO database (http://www.ncbi.nlm.nih.gov/geo) with the keywords “ischemia”[MeSH Terms] OR ischemic [All Fields]) AND “cardiomyopathies” [MeSH Terms] OR cardiomyopathy [All Fields]. Three series of datasets, GSE46224 [22], GSE52601 [23] and GSE5406 [24], were selected for data analyses according to the selection criteria described as follows: (1) Dataset should be whole-genome mRNA expression profile by array. (2) Datasets were obtained by left ventricular tissue samples of ICM and healthy control group. (3) The datasets should be normalized or original.

### Identification of DEGs

MetaMA, an R package, is applied to combine data from three GEO datasets. The Benjamini & Hochberg (False discovery rate; FDR) were used to modulate the *P*-values. The selection criteria for identification of DEGs were: FDR < 0.05. The R package was performed to produce the hierarchical clustering analysis of top 100 DEGs.

### Functional annotation

Gene Ontology (GO) classification and the Kyoto Encyclopedia of Genes and Genomes (KEGG) pathway enrichment analyses were structured by using GeneCodis (http://genecodis.cnb.csic.es/analysis). The terms with FDR < 0.05 was significant results.

### ICM -specific protein-protein interaction (PPI) network

The top 50 DEGs in ICM were applied to construct the PPI network by using Biological General Repository for Interaction Datasets (BioGRID) (http://thebiogrid.org/), and then the PPI network was visualized by Cytoscape (3.6.1) (http://www.cytoscape.org/). The nodes represent proteins and edges connect the nodes to show their relationship.

### ICM -specific transcriptional regulatory networks

The corresponding promoters of the top 20 up-regulated or down-regulated DEGs were obtained by UCSC (http://genome.ucsc.edu). The TF that regulates these DEGs comes from the matching tool in TRANSFAC. The ICM -specific transcriptional regulatory network was built by Cytoscape.

### Validation in the GEO dataset

The dataset of GSE116250 [25] was downloaded from the GEO database and used to validate the expression pattern of selected DEGs. The dataset GSE116250 was published on Nov 14, 2018 and examined the left ventricle tissue sample consisting of 13 ICM patients and 14 healthy controls.

### Confirmation by qRT-PCR

Patients presenting to Beijing Anzhen Hospital from July 2018 to December 2018 for coronary angiography were recruited consecutively for the study. Subjects were included in study as cases when left ventricular ejection fraction (LVEF) of ≤40% and fulfilling one of the following criteria: patients with history of myocardial infarction or revascularization (cardiac bypass surgery or percutaneous coronary intervention), patients with ≥75% stenosis of left main or proximal LAD, or patients with ≥75% stenosis of two or more epicardial vessels [26]. Subjects with LVEF of > 50 and < 50% stenosis in any main coronary artery were included as controls.

Ten patients diagnosed as ICM and 10 controls were enrolled in this study. The detailed characteristics of the patients were listed in Table 1. All patients were first on an empty stomach for 12 h. Then, we collected the blood samples by venipuncture at 7:00–8:00 of the next morning. This study has been approved by the ethics institute of our hospital. The signed informed consents of all the participants were obtained. Total RNA was isolated with the total RNA kit (Invitrogen, China). Fast Quant RT Kit (Invitrogen, China) was utilized to produce the complementary DNA. Then we performed the qRT-PCR with the Super Real PreMix Plus SYBR Green (Invitrogen, USA) on ABI 7500 real-time PCR system. The amplification process was performed under the following conditions: 15 min at 95 °C followed by 40 cycles of 10 s at 95 °C, 30 s at 55 °C, 32 s at 72 °C, and 15 s at 95 °C, 60 s at 60 °C, 15 s extension at 95 °C. The 2 − ΔΔCt method was used to address the data. The PCR primers used are displayed in Table 2.

**Table 1:** Baseline clinical characteristics of subjects

Characteristics	ICM(***n*** = 10)^**1**^	Control (n = 10)^**1**^	***P*** value^**2**^
Gender			> 0.90
Female	5 (50%)	5 (50%)	
Male	5 (50%)	5 (50%)	
Age	60 (51,64)	59 (58,62)	0.80
TC	4.12 (3.83, 5.02)	4.00 (3.76,4.33)	0.70
TG	2 (1,3)	1 (1,2)	0.50
HDL	1.16 (0.78,1.24)	1.04 (0.82,1.26)	> 0.90
LDL	2.70 (2.02,3.14)	2.57 (1.77,2.87)	0.70
Hypertension	3 (30%)	6 (60%)	0.40
Diabetes	5 (50%)	2 (20%)	0.30
Hyperlipidemia	3 (30%)	6 (60%)	0.40
Smoking	7 (70%)	4 (40%)	0.40
Drinking	1 (10%)	2 (20%)	> 0.90
HCT	34 (31,41)	39 (36,42)	0.13
HB	110 (103,144)	134 (122,150)	0.08

1 Statistics presented: *n* (%); median (IQR)

2 Statistical tests performed: chi-square test of independence; Wilcoxon rank-sum test; Fisher’s exact test

*Abbreviations*: *HDL-C* high-density lipoprotein cholesterol, *LDL-C* low-density lipoprotein cholesterol, *TG* triglyceride, *TC* Total cholesterol, *HCT* haematocrit, *HB* hemoglobin

**Table 2:** Primer sequences used for real-time PCR

Primer name	Sequence
ACTB-F	CATGTACGTTGCTATCCAGGC
ACTB-R	CTCCTTAATGTCACGCACGAT
ATP1A1-F	CTGTGGATTGGAGCGATTCTT
ATP1A1-R	TTACAACGGCTGATAGCACCA
BCL2L1-F	GAGCTGGTGGTTGACTTTCTC
BCL2L1-R	TCCATCTCCGATTCAGTCCCT
FKBP5-F	ACCAAACGGAAAGGAGAGGGA
FKBP5-R	TCTTCCCGCTGCATTTTCTCC
RRS1-F	GGCATCCGTCCCAAGAAGAAG
RRS1-R	TTCTTGGCCTGAATCCGCTTG
PALLD-F	AAGAAGGCCAGTAGAACTGCT
PALLD-R	AAGCGAAGTTTTCGTTCCAGG
ECM2-F	ACAAGCTCTATCACGTCCCG
ECM2-R	ACGGTCCATGCCATCATCAG

## Results

### DEGs in ICM

**Fig. 1:**
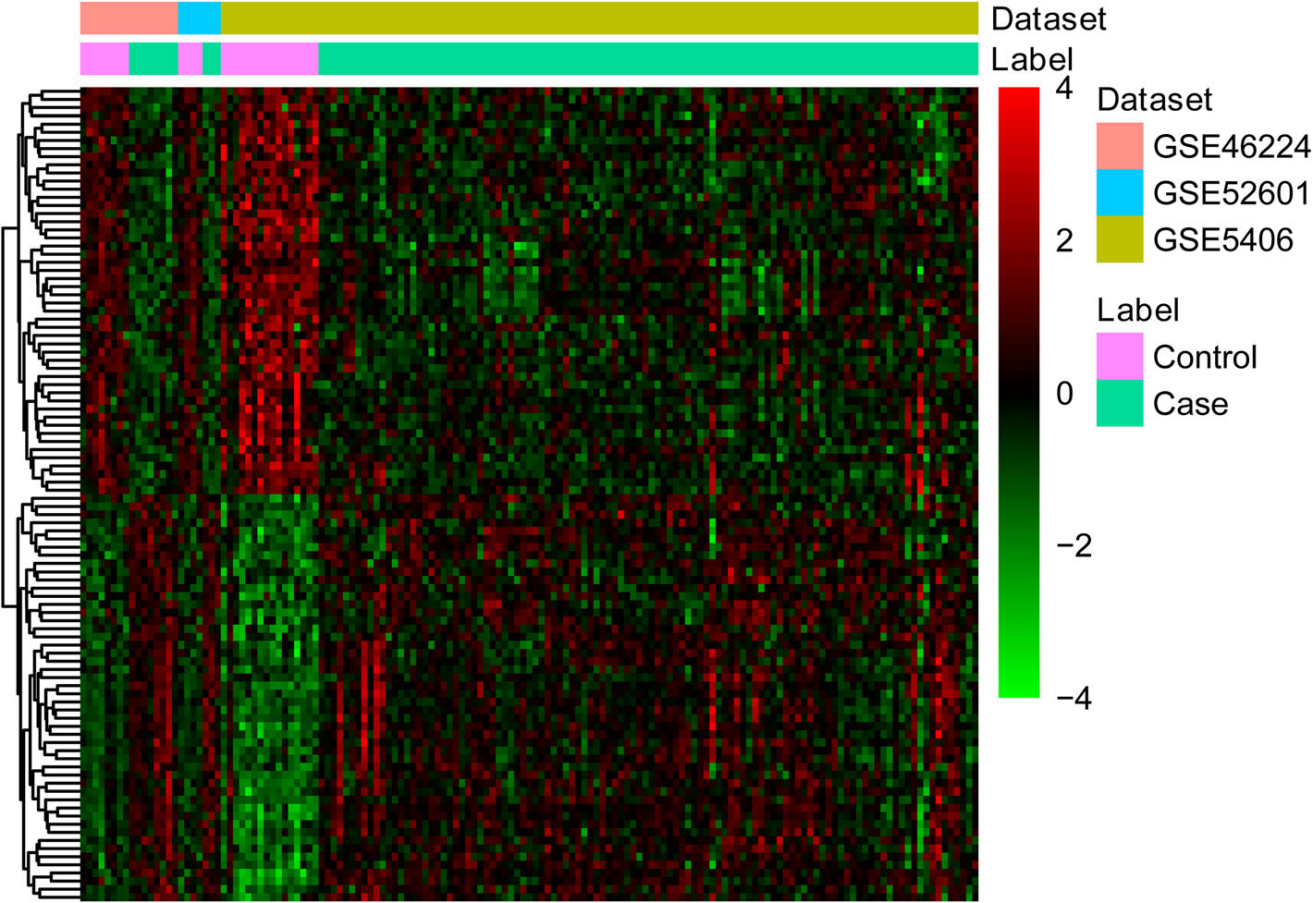
Heatmap of top 100 DEGs between ICM and healthy controls. Row and column represented DEGs and tissue samples, respectively. Color scale represented normalized expression levels of DEGs between ICM and healthy controls after log2 transformation. Red and green color were used to represent up- and downregulation in ICM, respectively

Three datasets (GSE46224, GSE52601 and GSE5406) were obtained from GEO (Table 3). Compared with the healthy controls, 1081 DEGs (578 genes were up-regulated and 503 genes were down-regulated) in ICM were obtained. All DEGs between ICM and healthy controls were displayed in Supplementary Table S1. Top 40 DEGs between ICM and healthy controls were demonstrated in Table 4. Hierarchical clustering of top 100 DEGs was indicated in Fig. 1.

**Table 3:** Gene expression datasets used in this study

GEO accession	Author	Platform	Samples (N:ICM)	Year	Tissue
GSE46224	Kai-Chien Yang [22]	GPL11154Illumina HiSeq 2000 (*Homo sapiens*)	8:8	2014	Left ventricle apex tissue
GSE52601	Akat KM [23]	GPL10558Illumina HumanHT-12 V4.0 expression beadchip	4:3	2014	left ventricular myocardium
GSE5406	Cappola TP [24]	GPL96 [HG-U133A] Affymetrix Human Genome U133A Array	16:108	2006	Left ventricular myocardium

**Table 4:** The top 40 DEGs in ICM

ID	Symbol	*P*.Value	FDR	Regulation
3151	HMGN2	0	0	Up
4060	LUM	0	0	Up
54,829	ASPN	0	0	Up
57,570	TRMT5	0	0	Up
5654	HTRA1	0	0	Up
25,878	MXRA5	0	0	Up
392	ARHGAP1	1.55E-15	1.30E-12	Up
2487	FRZB	3.19E-13	2.08E-10	Up
51,466	EVL	3.92E-13	2.30E-10	Up
4628	MYH10	1.08E-12	5.29E-10	Up
10,947	AP3M2	1.27E-12	5.98E-10	Up
3910	LAMA4	1.45E-12	6.30E-10	Up
3043	HBB	1.56E-12	6.54E-10	Up
1513	CTSK	4.13E-12	1.56E-09	Up
1842	ECM2	1.09E-11	3.86E-09	Up
4330	MN1	1.13E-11	3.92E-09	Up
7060	THBS4	1.63E-11	5.45E-09	Up
23,114	NFASC	3.06E-11	9.22E-09	Up
57,758	SCUBE2	5.44E-11	1.56E-08	Up
4878	NPPA	5.95E-11	1.66E-08	Up
8547	FCN3	0	0	Down
3163	HMOX2	0	0	Down
12	SERPINA3	0	0	Down
28,231	SLCO4A1	0	0	Down
4140	MARK3	0	0	Down
2289	FKBP5	4.44E-16	4.01E-13	Down
84,525	HOPX	1.40E-13	9.69E-11	Down
2752	GLUL	3.79E-13	2.30E-10	Down
476	ATP1A1	4.67E-13	2.45E-10	Down
262	AMD1	4.50E-13	2.45E-10	Down
10,157	AASS	1.42E-12	6.30E-10	Down
27,254	CSDC2	3.51E-12	1.42E-09	Down
4624	MYH6	4.11E-12	1.56E-09	Down
23,022	PALLD	6.10E-12	2.24E-09	Down
9057	SLC7A6	2.05E-11	6.68E-09	Down
9588	PRDX6	2.31E-11	7.33E-09	Down
598	BCL2L1	2.91E-11	9.00E-09	Down
5533	PPP3CC	3.50E-11	1.03E-08	Down
5604	MAP2K1	7.18E-11	1.92E-08	Down
23,212	RRS1	1.44E-10	3.53E-08	Down

### Functional annotation

**Fig. 2:**
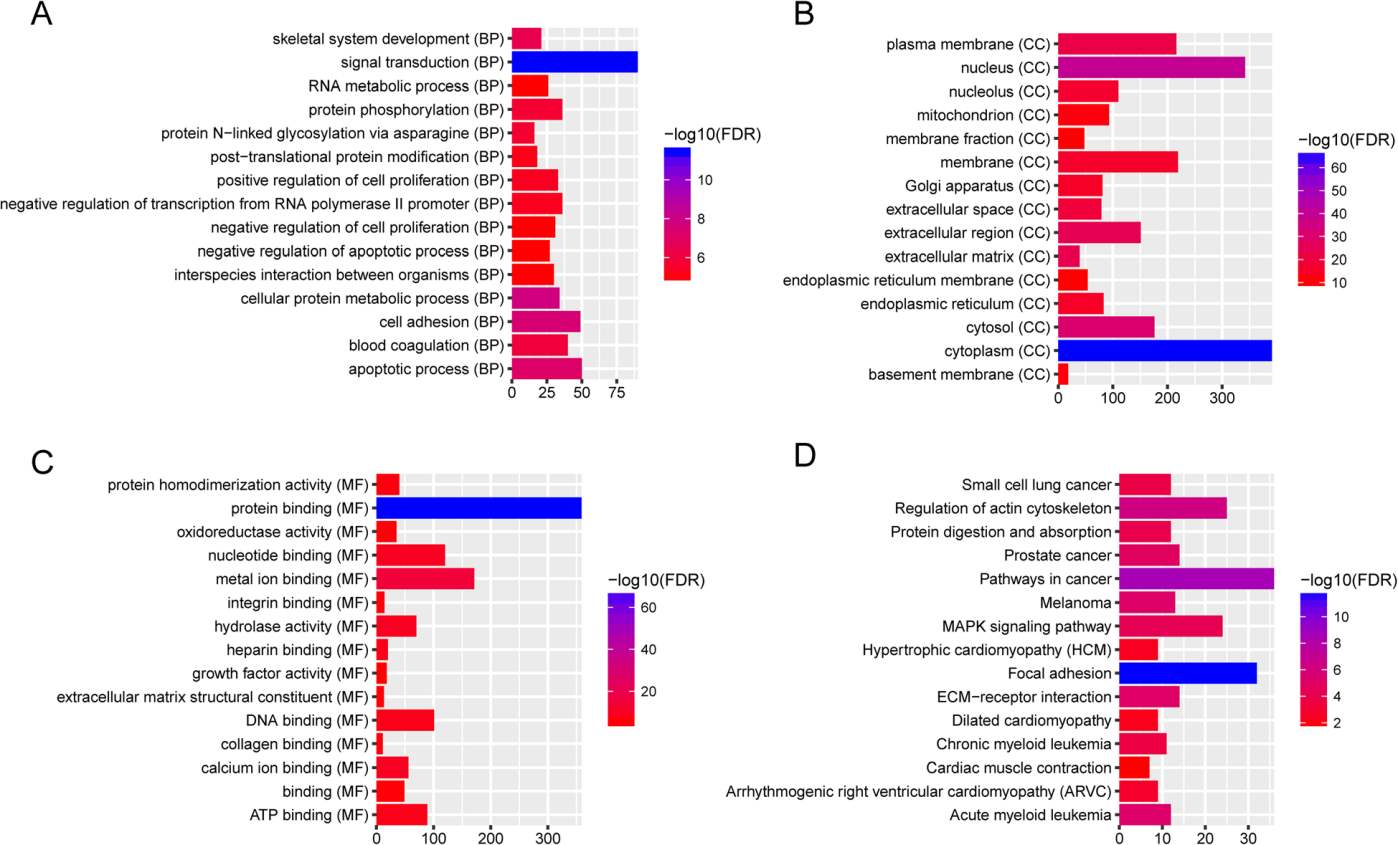
Top 15 most significantly enriched GO terms and KEGG pathways of DEGs in ICM. **a** Biological process. **b** Cellular component. **c** Molecular function. **d** KEGG pathways. The x-axis shows -log FDR and y-axis shows GO terms or KEGG pathways

**Fig. 3:**
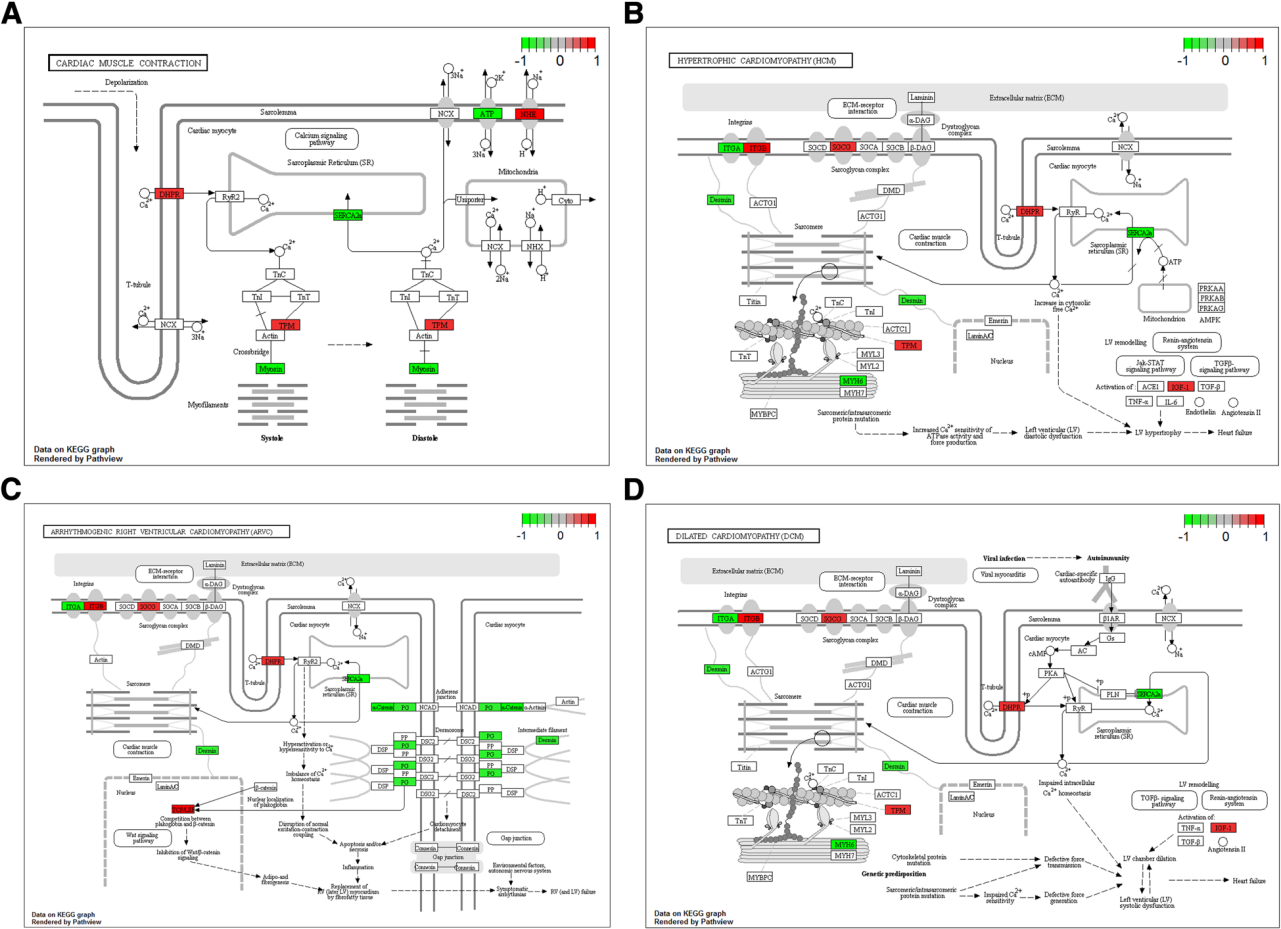
The key pathway of DEGs enrichment. a The cardiac muscle contraction pathway. **b** The hypertrophic cardiomyopathy pathway. **c**The arrhythmogenic right ventricular cardiomyopathy pathway. **d** The dilated cardiomyopathy pathway. The red rectangles were represented the components regulated by the DEGs that enriched in ICM

Base on the GO enrichment analysis, signal transduction (FDR = 3.04E-12), apoptotic process (FDR = 7.64E-08), cytoplasm (FDR = 3.40E-67) and protein binding (FDR = 6.06E-70) were most significantly enriched GO terms. After the KEGG pathway enrichment analysis, we found that ECM-receptor interaction (FDR = 8.23E-06), MAPK signaling pathway (FDR = 4.96E-05), Cardiac muscle contraction (FDR = 0.021418), hypertrophic cardiomyopathy (FDR = 0.003907), arrhythmogenic right ventricular cardiomyopathy (FDR = 0.00204) and dilated cardiomyopathy (FDR = 0.005789) were significantly enriched pathways in ICM. The top 15 most significantly enriched GO terms and KEGG pathways of DEGs in ICM were listed in Fig. 2 a-d. Pathways of cardiac muscle contraction, hypertrophic cardiomyopathy, arrhythmogenic right ventricular cardiomyopathy and dilated cardiomyopathy were displayed in Fig. 3a, b, c and d, respectively.

### ICM -specific PPI network

**Fig. 4:**
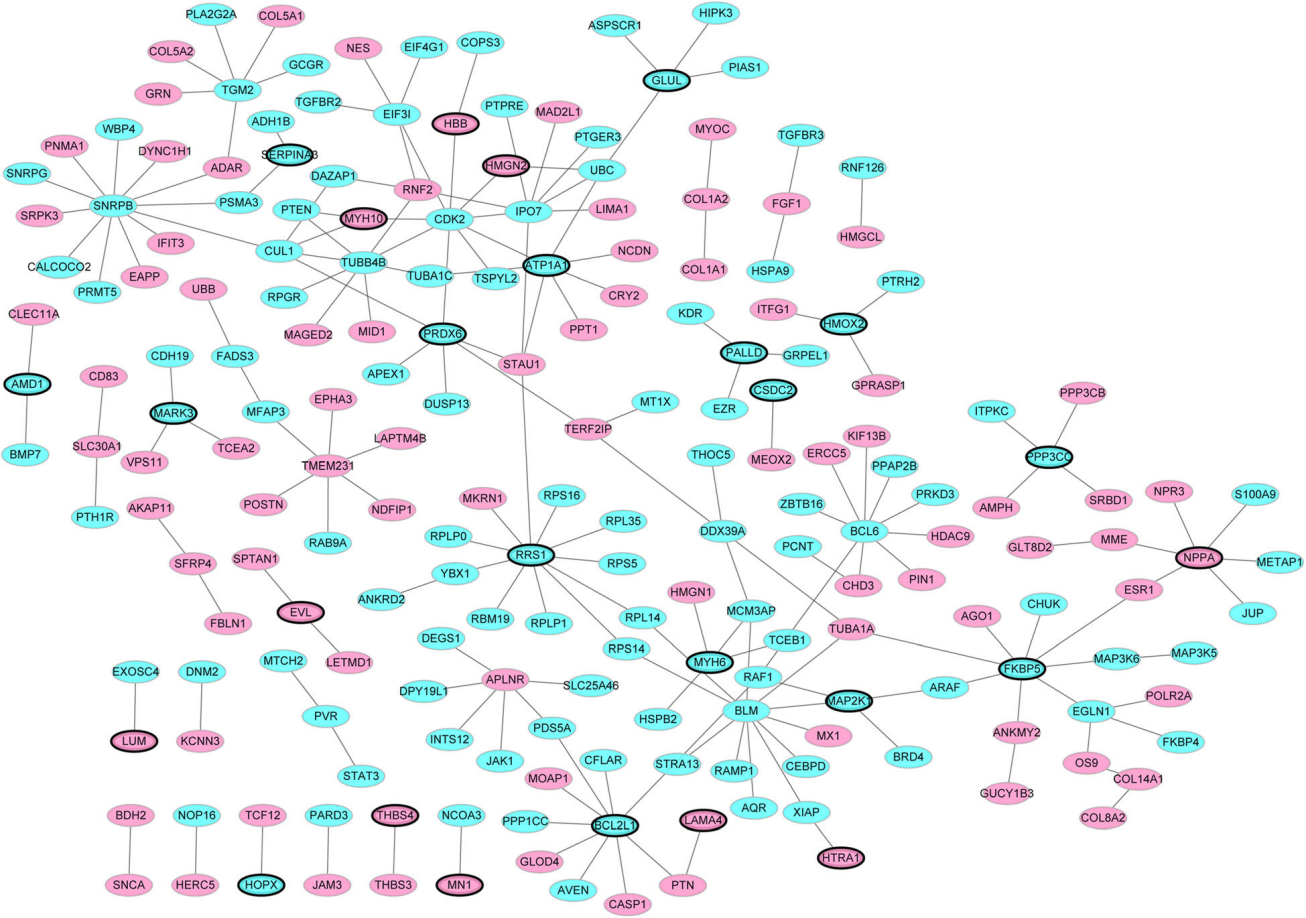
The ICM-specific PPI network. Ellipses were used to represent nodes and lines were used to represent edges. Green represents a downward adjustment and red represents a downward adjustment. The black border indicates top20Up/Down

The PPI network of top 50 DEGs in ICM was consisted of 208 nodes and 194 edges (Fig. 4). SNRPB (degree = 12), BLM (degree = 11), RRS1 (degree = 11), CDK2 (degree = 9), BCL6 (degree = 9), BCL2L1 (degree = 9), FKBP5 (degree = 8), IPO7 (degree = 8), TUBB4B (degree = 8) and ATP1A1 (degree = 7) were considered the hub proteins.

### ICM -specific transcriptional regulatory networks

**Fig. 5:**
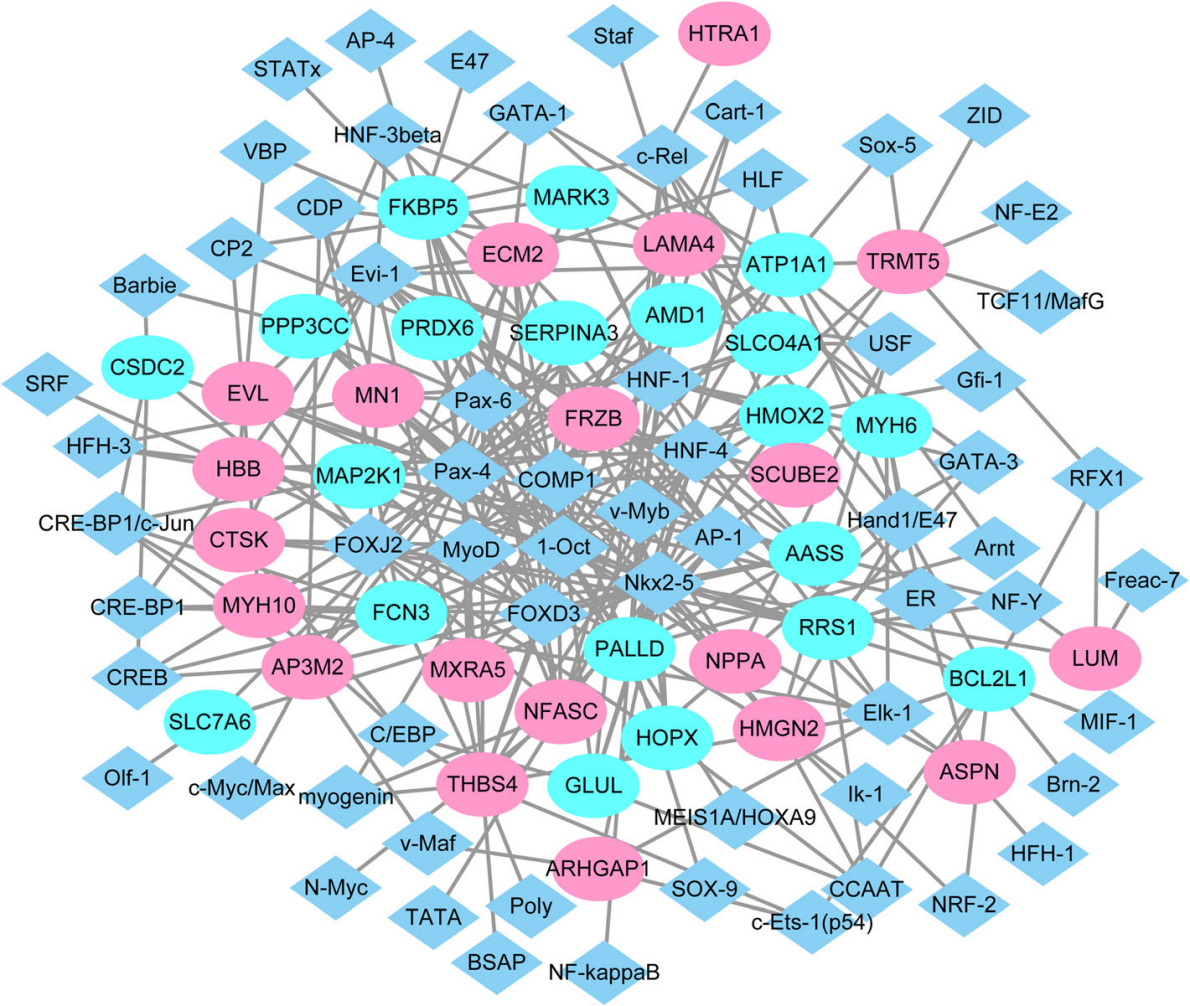
ICM -specific transcriptional regulatory network. Ellipses and rhombus were used to represent nodes and lines were used to represent edges. The rhombus represented DEGs and the ellipses represented TFs. Green represents a downward adjustment and red represents a downward adjustment. The lines indicated TFs-DEGs pairs

According to TRANSFAC, 64 TFs targeting 40 DEGs (top 20 up-regulated or down-regulated genes) were identified. ICM-specific transcriptional regulatory network was built, which consisted of 104 nodes and 290 edges (Fig. 5). Among of them, PALLD (degree = 17), THBS4 (degree = 14), ATP1A1 (degree = 12), NFASC (degree = 12), FKBP5 (degree = 12), ECM2 (degree = 12) and BCL2L1 (degree = 10) were top 6 TFs with the most downstream DEGs.

### Validation in GSE116250

**Fig. 6:**
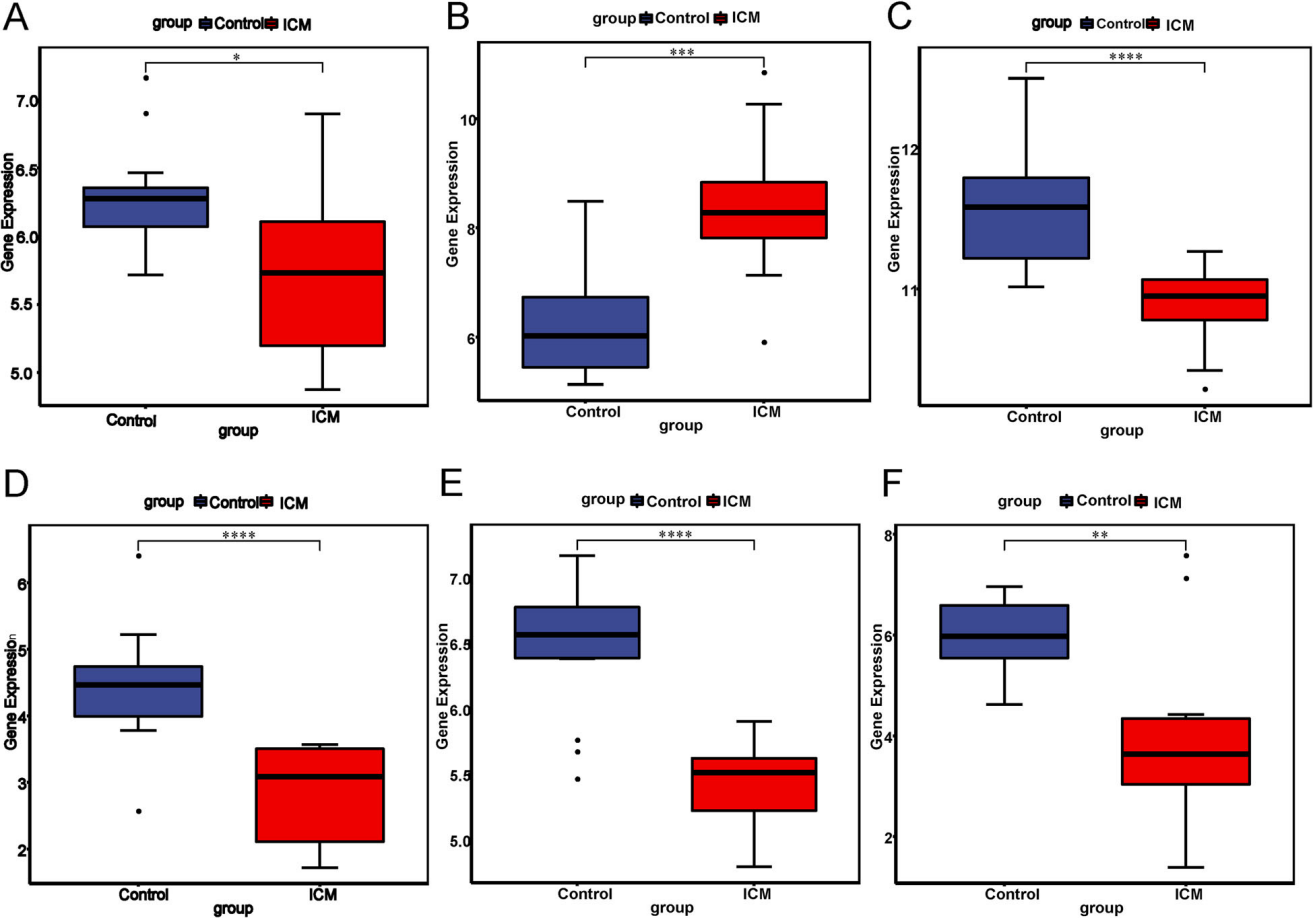
Validation of selected DEGs in GSE116250. a MYH6. **b** THBS4. **c** BCL6. **d** BLM. **e** IPO7. **f** SERPINA3. The x-axis shows healthy normal control (blue colour) and ICM (red colour) groups and y-axis shows a log2 transformation to the intensities. *P < 0.05, **P < 0.01, ***P < 0.001, ****P < 0.0001

Six DEGs (MYH6, THBS4, BCL6, BLM, IPO7 and SERPINA3) were selected to verify in GSE116250 dataset. Among them, BCL6, BLM and IPO7 were the hub gene of ICM-specific PPI network. THBS4 was top TFs covering the most downstream DEGs. MYH6 and SERPINA3 were top 40 DEGs in ICM. As displayed in Fig. 6, the expression of six DEGs were consistent with our integration results. MYH6, BCL6, BLM, IPO7 and SERPINA3 were down-regulated while THBS4 was up-regulated in ICM compared with healthy control.

### Validation by qRT-PCR

**Fig. 7:**
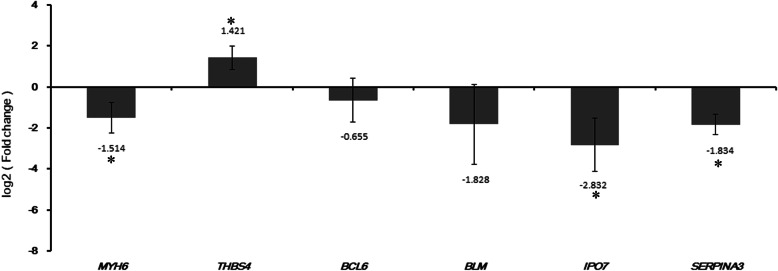
Validation DEG by qRT-PCR. The expression of DEG were detected by qRT-PCR assay. All of the assays were performed three times independently at least. Statistical significance was assessed by Student’s t-test. *P < 0.05

Six DEGs validated in GSE116250 were chose for qRT-PCR verification (Fig. 6). As shown in Fig. 7, MYH6, BCL6, BLM, IPO7 and SERPINA3 were down-regulated and THBS4 was up-regulated in ICM compared with control. In generally, the validation results of qRT-PCR were consistent with our integration results and GSE116250 validation results.

## Discussion

ICM continues to be one of the major diseases that threaten human health [1]. To make an accurate mechanism and find more effective therapeutic strategy for ICM in the early stage, it is need to find new therapeutic targets for ICM. With the emergence of high-throughput microarrays, a number of public resources have been build, among which the national center for biotechnology information (NCBI) GEO is the largest public resource [27]. Bioinformatics analysis based on GEO database provides valuable basis for revealing the pathogenesis of multiple diseases [28–30]. Integrated microarrays analysis with different platforms will obtain genome-wide expression profiling with larger sample size which will increase the statistical power than an individual microarray. Investigating abnormal gene expression in upstream TFs -mediated disease states can help to reveal the pathophysiological changes of complex diseases [31]. In the study, we carried out the integrated analysis of three gene expression datasets to identify the DEGs associated with ICM. A 1081 DEGs were identified in ICM with FDR < 0.05. The ICM related pathways and TFs were also obtained by the bioinformatics methods. We selected MYH6, THBS4, BCL6, BLM, IPO7 and SERPINA3 to verify their expression in ICM. Expression of 6 DEGs (MYH6, THBS4, BCL6, BLM, IPO7 and SERPINA3) in qRT-PCR results were consistent with our GEO analysis, which adds evidence to the reliability of our results.

MYH6 encodes the alpha heavy chain subunit of cardiac myosin in the developing atria. It has been reported that mutations of MYH6 associated with hypertrophic and dilated cardiomyopathy [32, 33]. MYH6 was associated with congenital heart disease, and indicate that by increase mutation of MYH6 could be associated with congenital heart disease [34]. Mutations in the head domain of MYH6 play a pivotal role in the progress of familial secundum-type atrial septal defects [35]. Jiang et al. found that silencing of mutant MYH6 transcripts in mice inhibited hypertrophic cardiomyopathy [36]. Castellana et al. reported the desmoglein-2/desmocollin-2/MYH6 mutations might determine a mild hypertrophic phenotype associated both to ventricular tachyarrhythmias and atrio-ventricular block [37]. Granados-Riveron et al. reported that mutations of MYH6 affecting myofibril formation are associated with congenital heart defects, whereas others have identified mutations of the same gene in patients with hypertrophic and dilated cardiomyopathy [38]. Here, MYH6 was down-regulated in patient with ICM in both integration analysis and qRT-PCR confirmation. The KEGG pathway enrichment analyses results showed that MYH6 was significantly enriched pathway of cardiac muscle contraction, hypertrophic cardiomyopathy and dilated cardiomyopathy. Therefore, we hypothesized that MYH6 might play key roles in ICM via regulating signaling pathway of cardiac muscle contraction, hypertrophic cardiomyopathy and dilated cardiomyopathy.

THBS4 is one of the exocrine glycoproteins involved in wound healing and tissue remodeling via modulating the repair and remodeling of the extracellular matrix [39, 40]. It has been found that THBS4 is continually abnormally expressed in the multiple solid cancers [41–43]. Recent research has indicated that THBS4 is involved in severe hypertrophic cardiomyopathy and heart failure pathogenesis [44]. In this study, THBS4 was one of top 6 TFs covering the most downstream DEGs, and was up-regulated in both integration analysis and qRT-PCR confirmation. The results displayed that THBS4 may play a key role in the pathogenesis of ICM. SERPINA3, a protease inhibitor, belongs to the superfamily of serine protease inhibitors. SERPINA3 is an acute phase response gene that is up-regulated during inflammation [45]. Masanori et al. found that SERPINA3 may be novel diagnostic and pharmacological targets for heart failure [46]. SERPINA3 has been reported to be involved in the pathogenesis of myocardial ischemia-reperfusion injury [47]. Herein, SERPINA3 was one of top 40 DEGs, and was down-regulated in both integration analysis and qRT-PCR confirmation. Therefore, we hypothesized that SERPINA3 may be involved in the development of ICM.

However, this study has several limitations that need to be acknowledged. The small samples size (10 sample per group) for qRT-PCR confirmation might affect the quality of our results. Although the validation based on GSE116250 suggested that our qRT-PCR results were generally convincing, studies with larger sample size need to be conducted to confirm this conclusion. The identification of DEGs of ICM is a pilot study and further model systems or cell lines experiments are needed to reveal their biological functions in ICM.

## Conclusions

The functional annotation, PPI network and ICM-specific transcriptional regulatory network were performed to identify DEGs, TFs and pathways in ICM which provides perspective to reveal the pathology and develop therapeutic targets for the ICM.

## Supplementary information

**Additional file 1.** All differentially expressed genes between ICM and healthy control.

## Data Availability

The datasets used and analysed during the current study are available from public database Gene Expression Omnibus repository. Accession numbers of the datasets used in current study are GSE46224, GSE52601, GSE5406 and GSE116250 in Gene Expression Omnibus (https://www.ncbi.nlm.nih.gov/geo).

## Abbreviations

ICM: Ischemic cardiomyopathy
DEGs: Differentially expressed genes
PPI: Protein-protein interaction
qRT-PCR: Quantitative real time polymerase chain reaction
GEO: Gene expression omnibus
TFs: Transcription factors
FDR: False discovery rate
GO: Gene ontology
KEGG: Kyoto encyclopedia of genes and genomes
BioGRID: Biological general repository for interaction datasets
NCBI: National center for biotechnology information
HDL-C: High-density lipoprotein cholesterol
LDL-C: Low-density lipoprotein cholesterol
TG: Triglyceride
TC: Total cholesterol
HCT: Haematocrit
HB: Hemoglobin
